# *In Silico* Investigation of Potential TRAF6 Inhibitor from Traditional Chinese Medicine against Cancers

**DOI:** 10.1155/2014/429486

**Published:** 2014-06-25

**Authors:** Kuan-Chung Chen, Wen-Yuan Lee, Hsin-Yi Chen, Calvin Yu-Chian Chen

**Affiliations:** ^1^School of Pharmacy, China Medical University, Taichung 40402, Taiwan; ^2^School of Medicine, College of Medicine, China Medical University, Taichung 40402, Taiwan; ^3^Department of Biomedical Informatics, Asia University, Taichung 41354, Taiwan; ^4^Department of Neurosurgery, China Medical University Hospital, Taichung 40447, Taiwan; ^5^Research Center for Chinese Medicine & Acupuncture, China Medical University, Taichung 40402, Taiwan; ^6^Human Genetic Center, Department of Medical Research, China Medical University Hospital, Taichung 40447, Taiwan

## Abstract

It has been indicated that tumor necrosis factor receptor-associated factor-6 (TRAF6) will upregulate the expression of hypoxia-inducible factor-1*α* (HIF-1*α*) and promote tumor angiogenesis. TRAF6 proteins can be treated as drug target proteins for a differentiation therapy against cancers. As structural disordered disposition in the protein may induce the side-effect and reduce the occupancy for ligand to bind with target protein, PONDR-Fit protocol was performed to predict the disordered disposition in TRAF6 protein before virtual screening. TCM compounds from the TCM Database@Taiwan were employed for virtual screening to identify potent compounds as lead compounds of TRAF6 inhibitor. After virtual screening, the MD simulation was performed to validate the stability of interactions between TRAF6 proteins and each ligand. The top TCM compounds, tryptophan, diiodotyrosine, and saussureamine C, extracted from *Saussurea lappa* Clarke, *Bos taurus domesticus* Gmelin, and *Lycium chinense* Mill., have higher binding affinities with target protein in docking simulation. However, the docking pose of TRAF6 protein with tryptophan is not stable under dynamic condition. For the other two TCM candidates, diiodotyrosine and saussureamine C maintain the similar docking poses under dynamic conditions. Hence, we propose the TCM compounds, diiodotyrosine and saussureamine C, as potential candidates as lead compounds for further study in drug development process with the TRAF6 protein against cancer.

## 1. Introduction

Nowadays, based on the increasing number of researches which identify the mechanisms of diseases [1–3], the number of potential target proteins for drug design against each disease is increasing sharply [4–6]. A recent research in cancer has indicated that tumor necrosis factor receptor-associated factor-6 (TRAF6) will promote tumor angiogenesis [7] as it will upregulate the expression of hypoxia-inducible factor-1*α* (HIF-1*α*) [8]. TRAF6 plays an important role in intracellular signal transduction as it can activate the function of NF-*κ*B [9, 10]. It belongs to a family of proteins which plays an important role in the regulation of inflammation, antiviral responses, and apoptosis [11, 12]. TRAF6 can also mediate the signaling from Toll/IL-1 family [13], CD40 [14, 15], and RANK [16]. Recent studies indicate that the overexpression of TRAF6 can induce a fatal acute myeloid leukemia [17] and several human cancer types [18, 19]. TRAF6 proteins can be treated as drug target proteins for a differentiation therapy against cancers.

The computer-aided virtual drug screening had been wildly used in the drug design [20, 21]. Many compounds from traditional Chinese medicine (TCM) have been identified as potential lead compounds for drug design against cancers [22–24], inflammation [25], influenza [26], viral infection, metabolic syndrome [27], diabetes [28], stroke [29–31], and many other diseases [32, 33]. To improve the development of TCM compounds, we aim to identify potent TCM compounds from the TCM Database@Taiwan [34] as lead compounds of TRAF6 inhibitor. As structural disordered disposition in the protein may induce the side-effect and reduce the occupancy for ligand to bind with target protein [35, 36], PONDR-Fit protocol was performed to predict the disordered disposition in TRAF6 protein before virtual screening. After virtual screening, the MD simulation was performed to validate the stability of interactions between TRAF6 proteins and each ligand.

## 2. Materials and Methods

### 2.1. Data Collection

The X-ray crystallography structure of the human TNF receptor-associated factor-6 (TRAF6) downloaded from RCSB Protein Data Bank with PDB ID 3HCT [37] was employed for virtual screening. PONDR-Fit [38] protocol was employed with the sequence of TRAF6 protein from Swiss-Prot (UniProtKB: Q9Y4K3) to predict the disordered amino acids. In preparation section, Prepare Protein module in Discovery Studio 2.5 (DS 2.5) was employed to protonate the X-ray crystallography structure of TRAF6 protein with Chemistry at HARvard Macromolecular Mechanics (CHARMM) force field [39] and remove crystal water. Prepare Ligand module in DS 2.5 was employed to protonate the final structure of TCM compounds from TCM Database@Taiwan [34] and filter TCM compounds using Lipinski's Rule of Five [40]. The binding site for virtual screening was defined closed to key residues Glu69, Pro71, Ile72, Leu74, Met75, Ala101, and Pro106.

### 2.2. Docking Simulation

LigandFit protocol [41] in DS 2.5 was employed using a shape filter and Monte-Carlo ligand conformation generation to dock the TCM compounds into the binding site. The protocol optionally minimized the docking poses with CHARMM force field [39] and filtered the similar poses using the clustering algorithm. Dock Score energy function was employed to evaluate the docking poses using the following equation:

(1) Dock Score=−(ligand receptor interaction energy  + ligand internal energy).

### 2.3. Molecular Dynamics (MD) Simulation

Gromacs 4.5.5 [42] was employed for the molecular dynamics (MD) simulation using classical molecular dynamics theory, which simulates each protein-ligand complex under dynamic conditions. In preparation section, the pdb2gmx protocol of Gromacs and SwissParam program [43] were employed to provide topology and parameters for TRAF6 proteins and each ligand, respectively. For solvation with TIP3P water model, the Gromacs protocol defined a cubic box upon the edge approximately 1.2 nm from the protein complexes periphery and creates a neutral system using 0.145 M NaCl model. A maximum of 5,000 steps of steepest descents [44] minimization was employed to remove bad van der Waals contacts. In equilibration section, a position-restrained molecular dynamics with the linear constraint algorithm for all bonds was performed using NVT equilibration, Berendsen weak thermal coupling method, and Particle Mesh Ewald method. A total of 10 ns production simulations were performed with time step in unit of 2 fs under NPT ensembles and Particle Mesh Ewald (PME) option. The 10 ns MD trajectories were then analyzed by a series of Gromacs protocols, and the presumable pathways for small molecule under dynamic conditions were analyzed by the CAVER 3.0 program [45].

## 3. Results and Discussion

### 3.1. Disordered Protein Prediction

**Figure 1:**
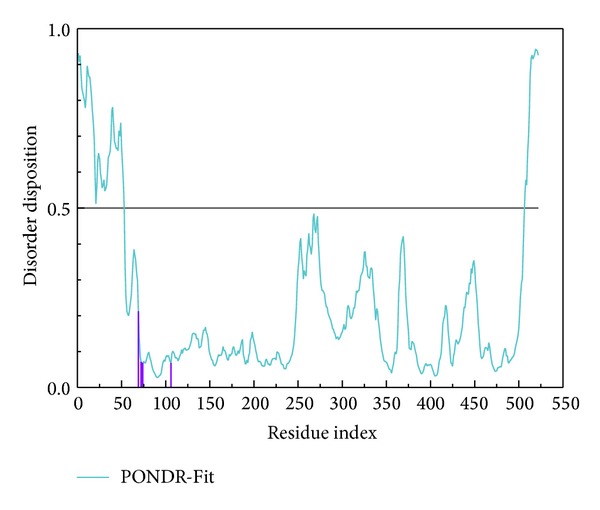
Disordered disposition predicted by PONDR-Fit. The residues in the binding domain are illustrated in purple lines.

The disordered disposition of TRAF6 protein was predicted by PONDR-Fit protocol [38] with the sequence from Swiss-Prot (UniProtKB: Q9Y4K3). The result displayed in Figure 1 indicates that the key residues in the binding domain do not lie in disordered disposition and they can form a stable binding domain in protein folding. As the residues in the binding domain have no significant variation, the crystallography structure of TRAF6 protein is a suitable receptor for docking simulation.

### 3.2. Docking Simulation

**Figure 2:**
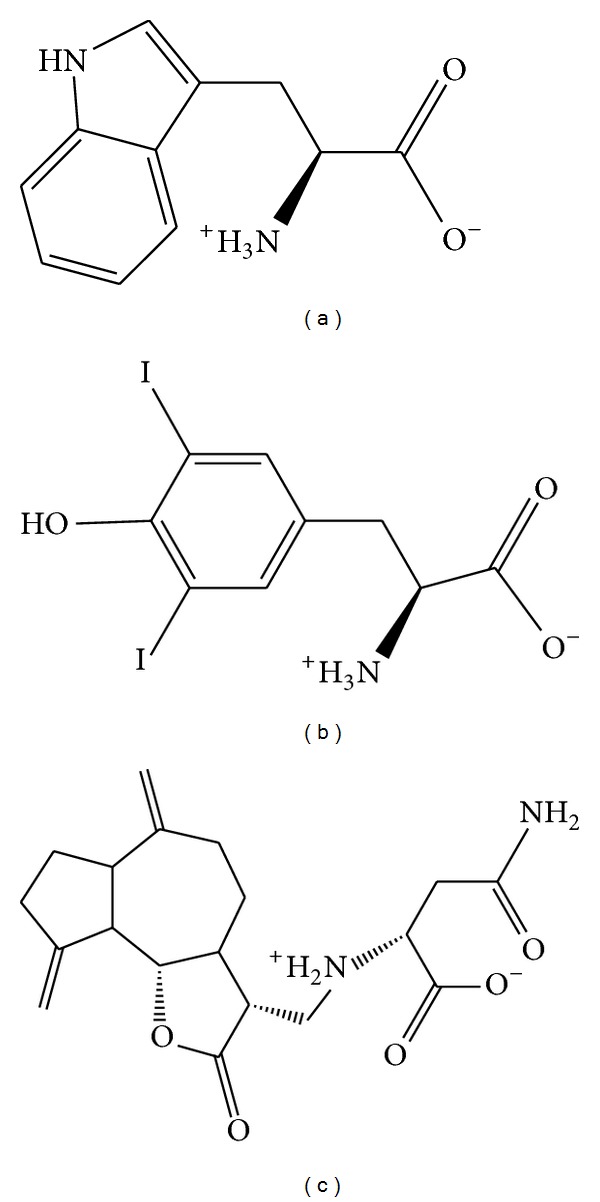
Chemical scaffold of top three TCM candidates. (a) Tryptophan, (b) diiodotyrosine, and (c) saussureamine C.

**Figure 3:**
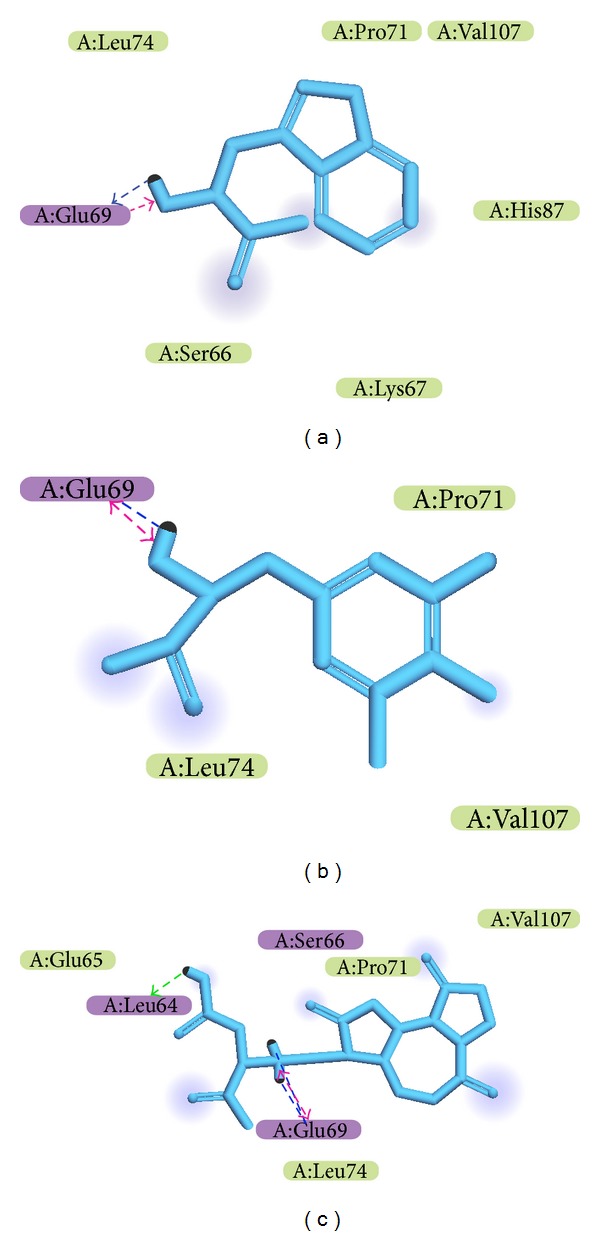
Docking pose of TRAF6 protein complex with (a) tryptophan, (b) diiodotyrosine, and (c) saussureamine C.

**Figure 4:**
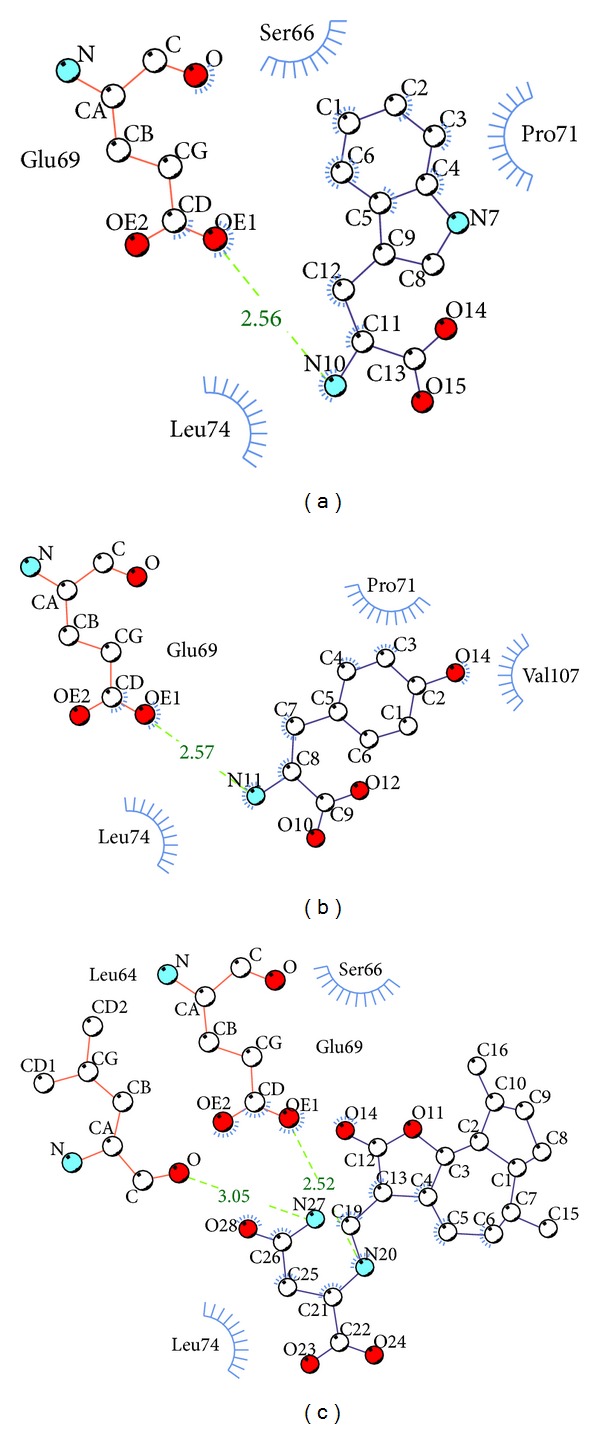
Docking pose of TRAF6 protein complex with (a) tryptophan, (b) diiodotyrosine, and (c) saussureamine C drawn by LigPlot program.

After virtual screening, the chemical scaffold top TCM compounds ranked by Dock Score [41] are shown in Table 1 with the scoring function of -PLP1, -PLP2, and -PMF. For the top three TCM compounds, tryptophan, diiodotyrosine, and saussureamine C, which were extracted from* Saussurea lappa* Clarke,* Bos taurus domesticus* Gmelin, and* Lycium chinense* Mill., the chemical scaffold top TCM compounds are shown in Figure 2. According to the docking poses in Figure 3, the top three candidate compounds have interaction with common residue Glu69, and diiodotyrosine also has an H-bond with residue Leu64. In addition, the docking poses drawn by LigPlot program in Figure 4 showed that the residues Ser66, Pro71, and Leu74 are common residues in the binding domain to form the hydrophobic contacts with ligands.

### 3.3. Molecular Dynamics Simulation

**Figure 5:**
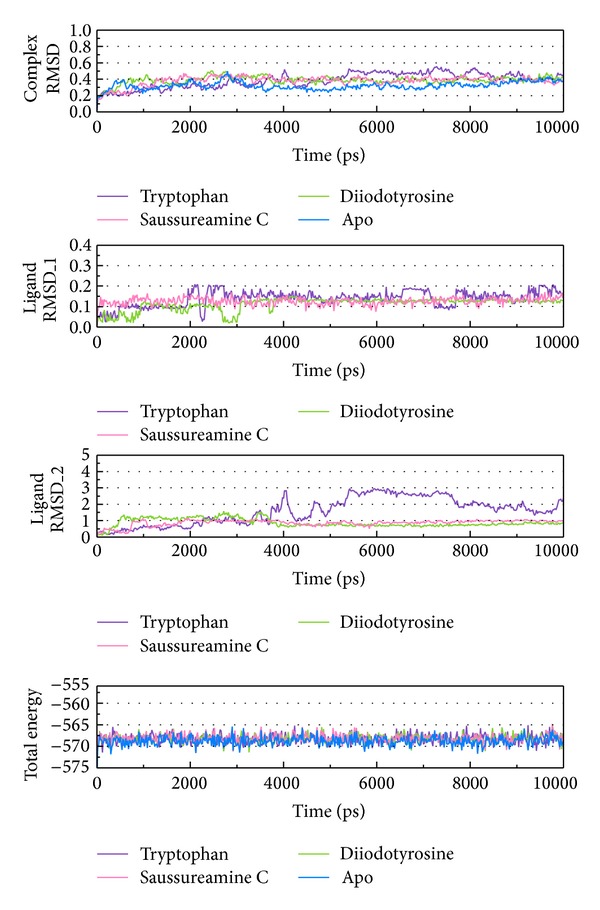
Root-mean-square deviations in units of nm for protein and ligand and variation of total energy in units of 10^3^ kJ/mole for TRAF6 proteins in apo form and complexes with tryptophan, diiodotyrosine, and saussureamine C over 10 ns of MD simulation.

**Figure 6:**
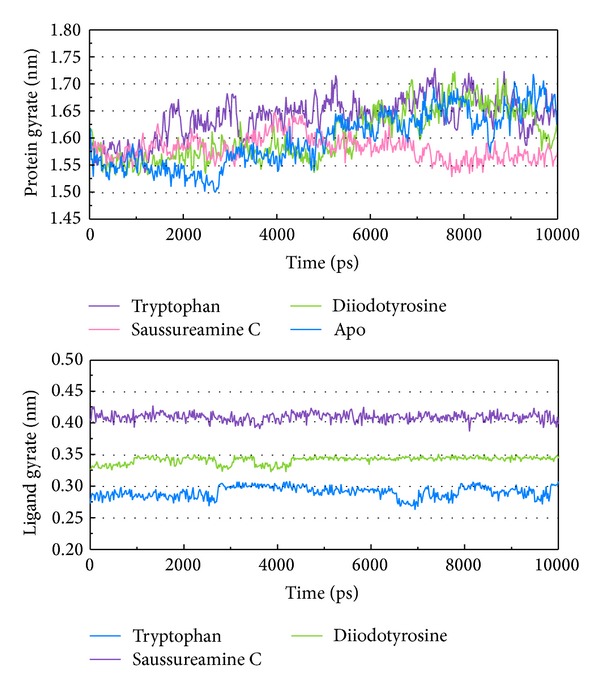
Radii of gyration for protein and ligands of TRAF6 proteins in apo form and complexes with tryptophan, diiodotyrosine, and saussureamine C over 10 ns of MD simulation.

**Figure 7:**
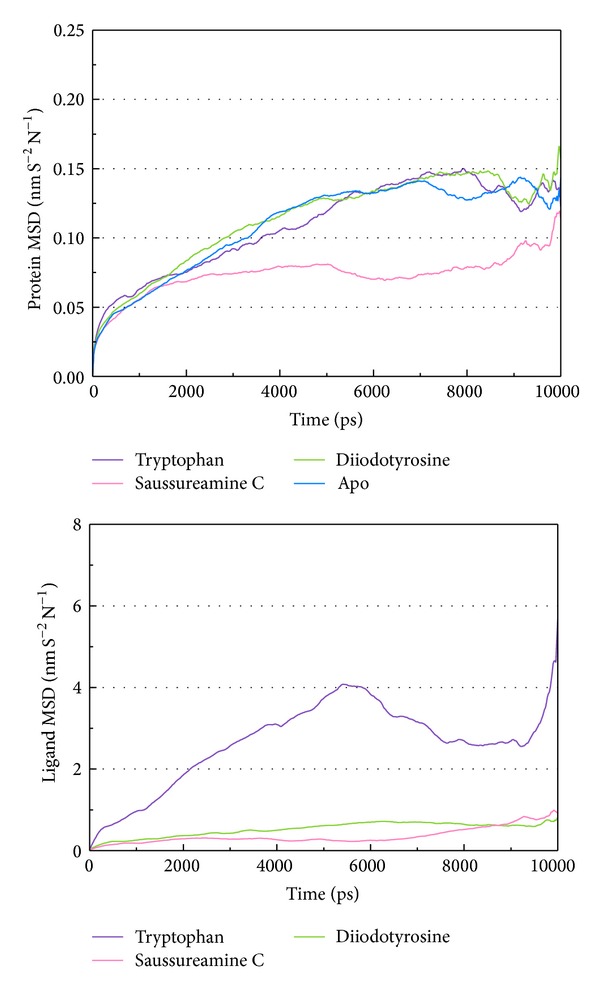
Mean square displacement (MSD) for protein and ligand over 10 ns of MD simulation for TRAF6 proteins in apo form and complexes with tryptophan, diiodotyrosine, and saussureamine C.

**Figure 8:**
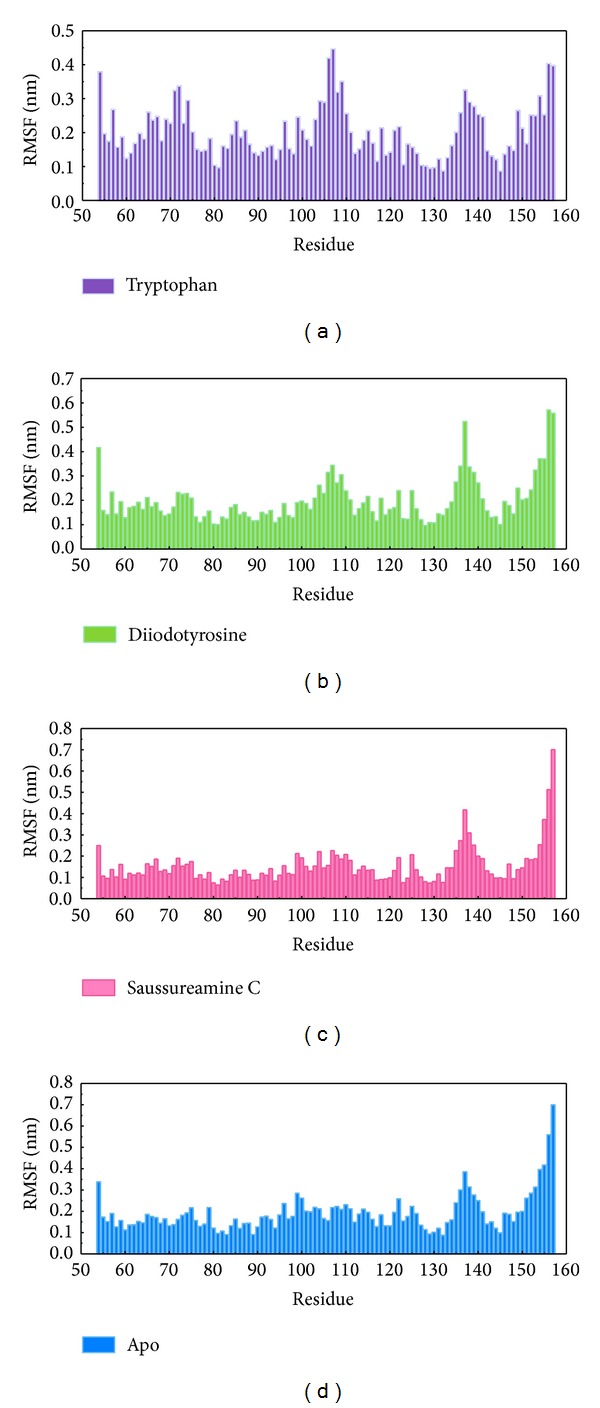
Root-mean-square fluctuation (RMSF) for residues of TRAF6 proteins in complexes with (a) tryptophan, (b) diiodotyrosine, and (c) saussureamine C and in (d) apo form.

**Figure 9:**
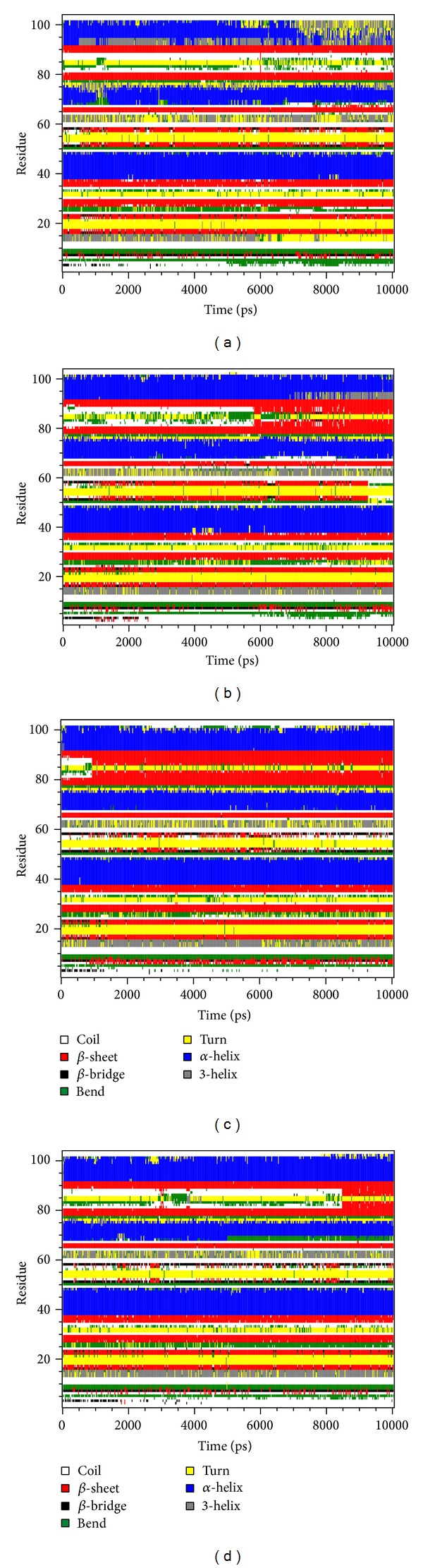
Variation of secondary structure of TRAF6 proteins in complexes with (a) tryptophan, (b) diiodotyrosine, and (c) saussureamine C and in (d) apo form.

As the TRAF6 proteins present as a rigid body in docking simulation performed by LigandFit protocol, the interactions between ligand and protein may be varied while the conformation of the TRAF6 protein was modified under dynamic conditions. The MD simulation was employed to validate the stability of interactions between TRAF6 proteins and each ligand. Root-mean-square deviation (RMSD) displayed the atomic fluctuations during MD simulation. The complex RMSD in Figure 5 indicates that the atomic fluctuations of TRAF6 proteins in apo form and complexes with tryptophan, diiodotyrosine, and saussureamine C tended to stabilize after 9000 ps of MD simulation. For the atomic fluctuations of each compound, the ligand RMSD_1 and RMSD_2 displayed the RMSD values of each ligand, which were calculated after the least squares fitting of protein and ligand, respectively. They indicate that the docking pose of tryptophan tends to destabilize after 3 ns of MD simulation. For the other TCM candidates, diiodotyrosine and saussureamine C, the structure of compounds tends to stabilize after 4 ns of MD simulation. In addition, the total energy over 10 ns MD simulation for each complex in Figure 5 indicates that there is no significant change for the total energies of each TRAF6 protein complex during MD simulation.  Figure 6 displayed the variation radii of gyration for protein and ligands over 10 ns of MD simulation. It indicates that the radii of gyration for TRAF6 proteins in complexes of TRAF6 with diiodotyrosine and saussureamine C were smaller than TRAF6 proteins in apo form and in complexes of TRAF6 protein with tryptophan after MD simulation, while the radii of gyration for each ligand were stabilized under dynamic condition. The mean square displacement (MSD) for protein and each ligand was illustrated in Figure 7. The protein MSD indicates that the diffusion constants of TRAF6 proteins in apo form and in each TRAF6 complex were similar after 10 ns of MD simulation. However, the ligand MSD indicates that the diffusion constant of atoms for tryptophan is increasing sharply after MD simulation. It indicates that docking pose of tryptophan has a rapidly variation under dynamic condition. In Figure 8, root-mean-square fluctuation (RMSF) for each residue over 10 ns of MD simulation indicates that the residues from Lys106 to Asn109 are more flexible in complexes of TRAF6 protein with tryptophan than in TRAF6 proteins in apo form and in complexes of TRAF6 protein with diiodotyrosine and saussureamine C. It indicates that TRAF6 proteins docking with diiodotyrosine and saussureamine C causes similar flexibility for TRAF6 proteins. In addition, Figure 9 illustrated the change of secondary structure of TRAF6 proteins in apo form and complexes with tryptophan, diiodotyrosine, and saussureamine C. There is no significant change in the secondary structure of TRAF6 proteins in apo form and each complex.

**Figure 10:**
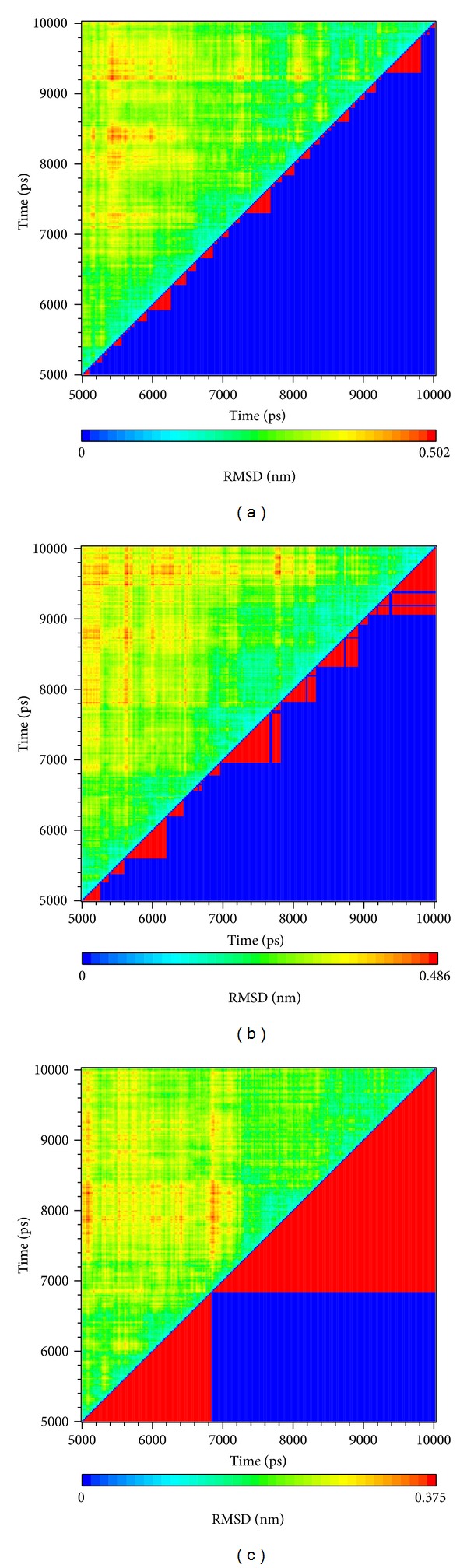
Root-mean-square deviation value (upper left half) and graphical depiction of the clusters with cutoff 0.14 nm (lower right half) for TRAF6 protein complexes with (a) tryptophan, (b) diiodotyrosine, and (c) saussureamine C.

**Figure 11:**
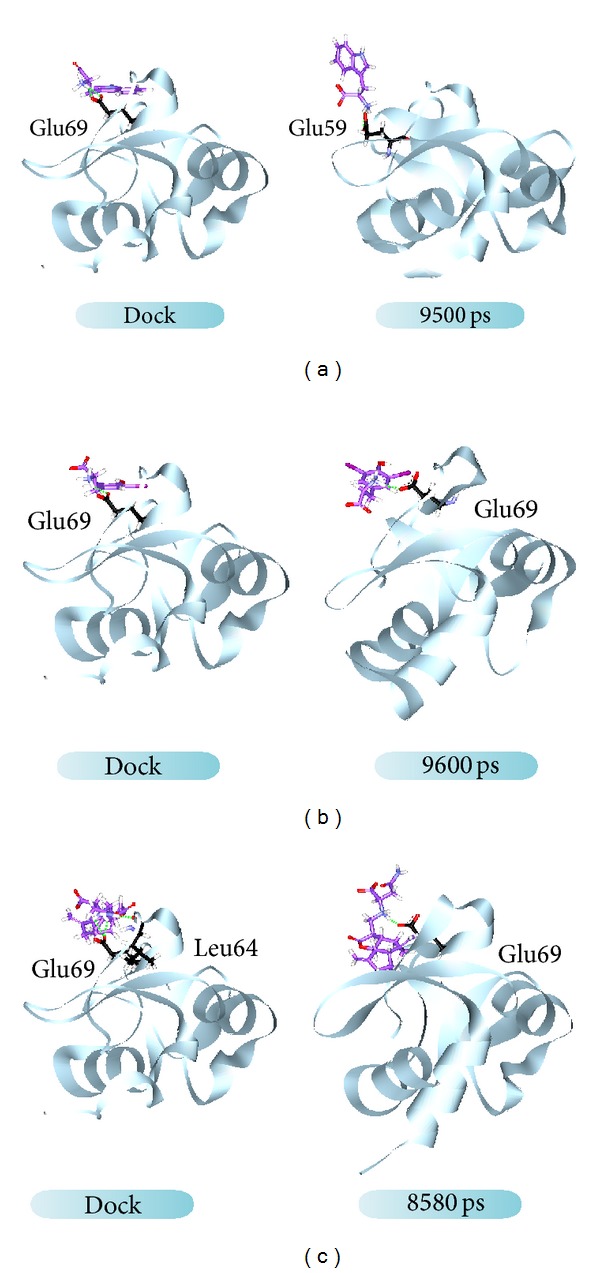
Docking poses in docking simulation and representative structures of TRAF6 protein complexes with (a) tryptophan, (b) diiodotyrosine, and (c) saussureamine C.

**Figure 12:**
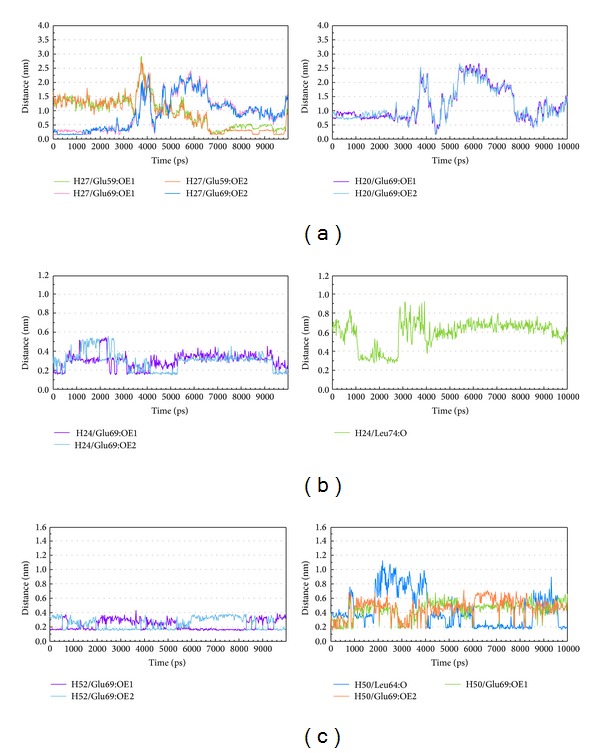
Distance variation of H-bonds with TRAF6 protein during MD simulation. (a) Tryptophan, (b) diiodotyrosine, and (c) saussureamine C.

**Figure 13:**
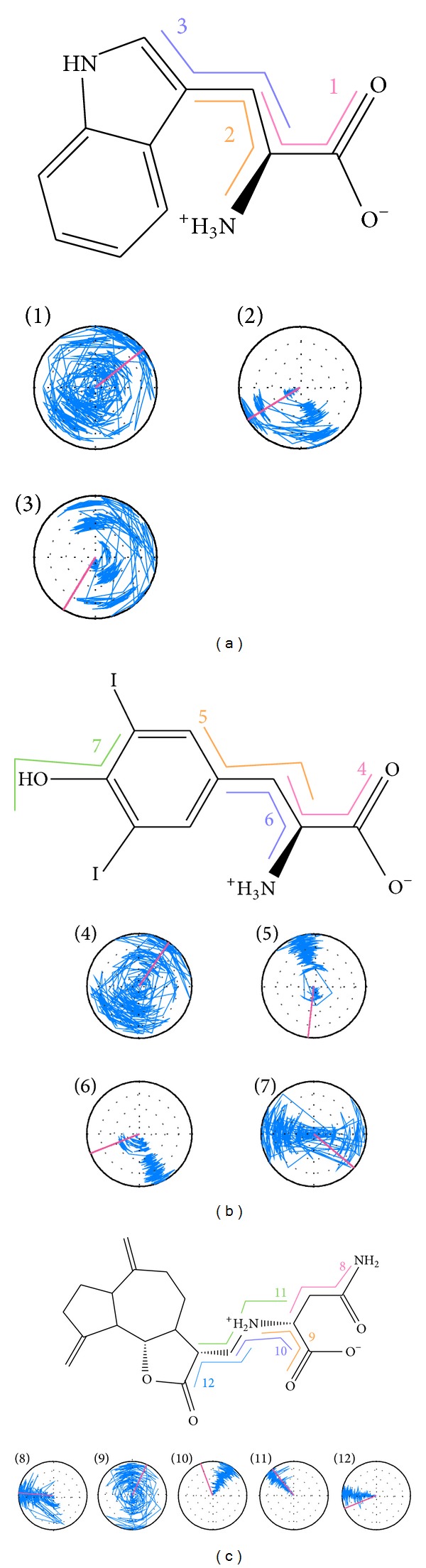
Dialplots of torsion angles for ligands during MD. The plots illustrate the time-dependent change of the dihedrals for (a) tryptophan, (b) diiodotyrosine, and (c) saussureamine C.

**Figure 14:**
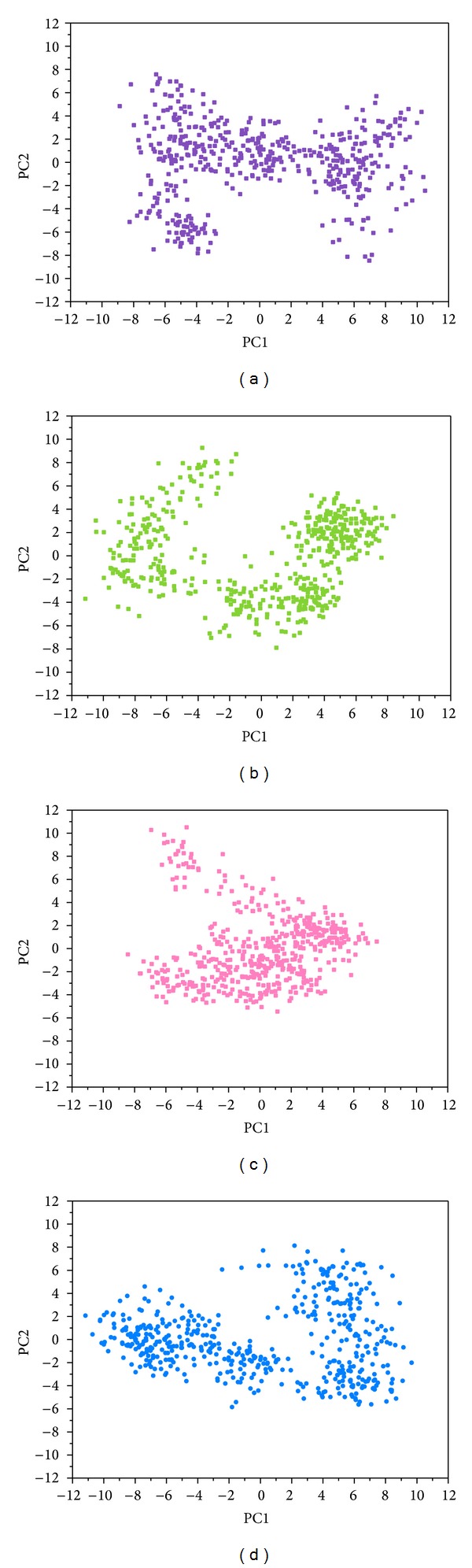
Eigenvector distribution of TRAF6 proteins in complexes with (a) tryptophan, (b) diiodotyrosine, and (c) saussureamine C and in (d) apo form.

**Figure 15:**
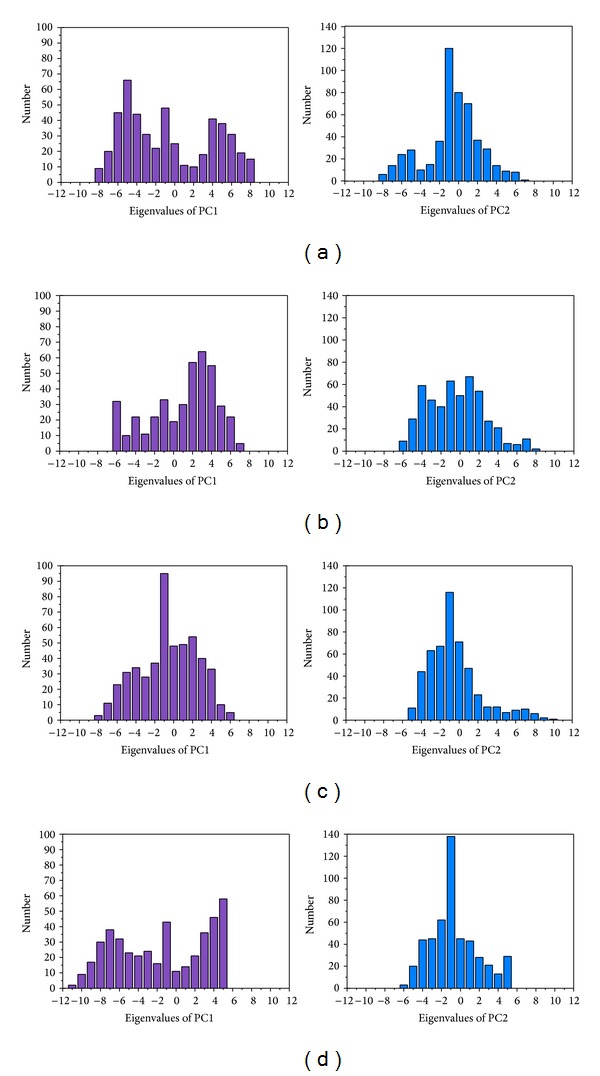
Distribution of Eigenvector PC1 and PC2 for TRAF6 proteins in complexes with (a) tryptophan, (b) diiodotyrosine, (c) and saussureamine C and in (d) apo form.

**Figure 16:**
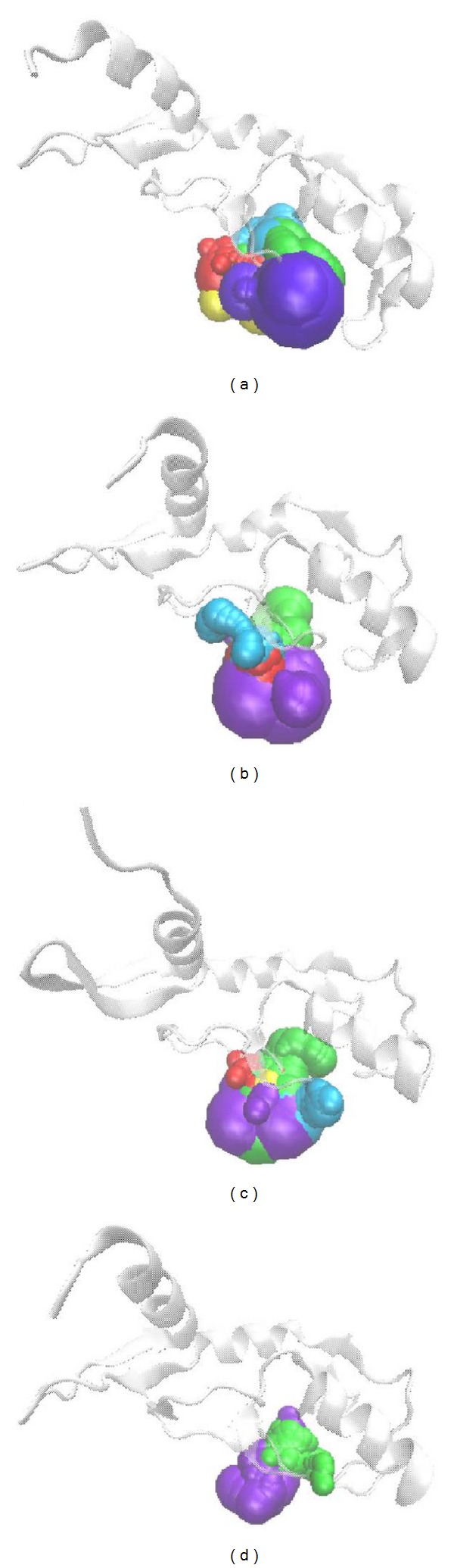
Analysis of transport pathways for TRAF6 proteins in complexes with (a) tryptophan, (b) diiodotyrosine, and (c) saussureamine C and in (d) apo form.

The RMSD values and graphical depiction of the clusters analysis with a RMSD cut-off of 0.14 nm during 5–10 ns of MD simulation in Figure 10 indicate the representative structures of TRAF6 protein complexes with tryptophan, diiodotyrosine, and saussureamine C. After MD simulation, the docking poses in docking simulation and representative structures of each TRAF6 protein complex are illustrated in Figure 11. The TCM candidates except tryptophan have similar docking poses as docking simulation, which has stable H-bonds with residues Glu69. For TRAF6 protein complex with tryptophan, the docking pose of tryptophan was changed. It misses out the H-bond with Glu69 and forms an H-bond with Glu59 after MD simulation. The H-bond occupancy for key residues in complexes of TRAF6 protein with top TCM compounds over 10 ns of MD simulation was listed in Table 2 and the distance variation of each H-bond was illustrated in Figure 12. For tryptophan, it cannot maintain the H-bonds with key residue Glu69 under dynamic condition. For diiodotyrosine and saussureamine C, they maintain the H-bonds with key residue Glu69 under dynamic condition. In addition, saussureamine C forms an H-bond with residue Leu64 after MD simulation.  Figure 13 illustrates the variation of torsion angles in each ligand over 10 ns of MD simulation. For tryptophan, the torsion angles 1 and 3 were messy as the docking pose in complexes of TRAF6 protein with tryptophan was varied during MD simulation. For diiodotyrosine, the torsion angles 5 and 6 tend to stabilize after a short period of MD simulation; the torsion angle 4 was fluctuated, as two oxygen atoms in carboxyl group own equal opportunity to form the H-bonds under dynamic condition. For saussureamine C, the torsion angles also tend to stabilize after a short period of MD simulation except that torsion angle 4 was fluctuated as two oxygen atoms in carboxyl group own equal opportunity to form the H-bonds under dynamic condition.  Figure 14 displays the projection of trajectories on eigenvectors 1 and 2 for TRAF6 proteins in apo form and complexes with tryptophan, diiodotyrosine, and saussureamine C.  Figure 15 illustrated the distribution of eigenvectors 1 and 2, respectively, for TRAF6 proteins in apo form and complexes with tryptophan, diiodotyrosine, and saussureamine C. They indicate that TRAF6 proteins in complexes with tryptophan has the larger fluctuation in both major eigenvectors than in the others. Analysis of transport pathways for each TRAF6 protein complex illustrated in Figure 16 indicates the presumable pathways for small molecule. They show that the TRAF6 protein complex has more potential pathway than that in apo form, which indicates that the space of binding domain has varied after docking with the TCM compounds.

## 4. Conclusion

This study aims to investigate the potent lead TCM candidates for TRAF6 protein inhibitors against cancers. The top TCM compounds, tryptophan, diiodotyrosine, and saussureamine C, have higher binding affinities with target protein in docking simulation. They has H-bonds with residues Glu69 and hydrophobic contacts with common residues Ser66, Pro71, and Leu74. After MD simulation, the top TCM compounds except tryptophan maintain the similar docking poses under dynamic conditions. For tryptophan, the docking pose has varied under dynamic condition and misses out the H-bond with Glu69 to form an H-bond with Glu59. For the other two TCM candidates, diiodotyrosine and saussureamine C were extracted from* Bos taurus domesticus *Gmelin and* Lycium chinense* Mill. Hence, we propose the TCM compounds, diiodotyrosine and saussureamine C, as potential candidates as lead compounds for further study in drug development process with the TRAF6 protein against cancers.

## Figures and Tables

**Table 1:** Scoring functions of top candidates from TCM database screening.

Name	Dock score	-PLP1	-PLP2	-PMF
Tryptophan	171.283	27.71	27.51	23.06
Diiodotyrosine	171.000	20.22	20.27	23.51
Saussureamine C	170.814	27.27	24.71	42.2
Eupachinilide J	170.774	32.62	27.11	25.13
Tryptophan	169.656	36.05	32.98	21.91
Diiodotyrosine	168.307	33.24	27.04	16.58
5-Hydroxy-L-tryptophan	166.948	30.73	32.06	27.43
L-Tyrosine	165.590	33.52	33.82	20.64
S-Allylmercaptocysteine	165.014	17.05	18.65	16.6
Emetine	162.586	29.04	27.44	8.71

**Table 2:** H-bond occupancy for key residues of TRAF6 protein with top three TCM candidates over 10 ns of MD simulation.

Ligand	H-bond	Ligand atom	Amino acid	Distance (nm)			Occupancy (%)
				Max.	Min.	Average	
Tryptophan	1	H27	Glu59:OE1	2.93	0.15	0.95	13.0%
	2	H27	Glu59:OE2	2.729	0.152	0.901	32.6%
	3	H20	Glu69:OE1	2.68	0.163	1.229	0.8%
	4	H20	Glu69:OE2	2.671	0.17	1.214	1.0%
	5	H27	Glu69:OE1	2.427	0.146	0.933	29.6%
	6	H27	Glu69:OE2	2.318	0.148	0.926	25.4%

Diiodotyrosine	1	H24	Glu69:OE1	0.55	0.15	0.30	77%
	2	H24	Glu69:OE2	0.56	0.15	0.30	82%
	3	H24	Leu74:O	0.92	0.27	0.59	14.2%

Saussureamine C	1	H50	Leu64:O	1.13	0.16	0.44	45.0%
	2	H50	Glu69:OE1	0.69	0.16	0.44	19.6%
	3	H50	Glu69:OE2	0.72	0.15	0.46	17.8%
	4	H52	Glu69:OE1	0.44	0.15	0.23	95.6%
	5	H52	Glu69:OE2	0.38	0.15	0.24	85.8%

H-bond occupancy cut-off is 0.35 nm.
